# Supplementary material for: Pyruvate Dehydrogenase Kinase 1 inhibition mediated oxidative phosphorylation enhancement in cartilage promotes osteoarthritis progression
Source: BMC Musculoskelet Disord. 2023 Jul 20;24:597. doi: 10.1186/s12891-023-06585-6 (PMC10357736; doi:10.1186/s12891-023-06585-6)
Supplement: Supplementary file 1 — Additional file 1. [file 12891_2023_6585_MOESM1_ESM.zip › KEGG.Gsea.1653622432464/KEGG_UBIQUITIN_MEDIATED_PROTEOLYSIS.html]

Details for gene set KEGG\_UBIQUITIN\_MEDIATED\_PROTEOLYSIS[GSEA]

|  || Dataset | 55457\_collapsed\_to\_symbols.cls.cls #OA\_versus\_normal.cls.cls #OA\_versus\_normal\_repos |
| Phenotype | cls.cls#OA\_versus\_normal\_repos |
| Upregulated in class | normal |
| GeneSet | KEGG\_UBIQUITIN\_MEDIATED\_PROTEOLYSIS |
| Enrichment Score (ES) | -0.17632745 |
| Normalized Enrichment Score (NES) | -0.7844143 |
| Nominal p-value | 0.90462 |
| FDR q-value | 0.97262716 |
| FWER p-Value | 1.0 |
Table: GSEA Results Summary

  

Fig 1: Enrichment plot: KEGG\_UBIQUITIN\_MEDIATED\_PROTEOLYSIS      
 Profile of the Running ES Score & Positions of GeneSet Members on the Rank Ordered List

  

| SYMBOL | TITLE | RANK IN GENE LIST | RANK METRIC SCORE | RUNNING ES | CORE ENRICHMENT || 1 | DET1 | DET1 partner of COP1 E3 ubiquitin ligase [Source:HGNC Symbol;Acc:HGNC:25477] | 50 | 0.989 | 0.0251 | Yes |
| 2 | UBE2E3 | ubiquitin conjugating enzyme E2 E3 [Source:HGNC Symbol;Acc:HGNC:12479] | 151 | 0.860 | 0.0424 | Yes |
| 3 | STUB1 | STIP1 homology and U-box containing protein 1 [Source:HGNC Symbol;Acc:HGNC:11427] | 345 | 0.726 | 0.0486 | Yes |
| 4 | RBX1 | ring-box 1 [Source:HGNC Symbol;Acc:HGNC:9928] | 348 | 0.724 | 0.0696 | Yes |
| 5 | UBE2D2 | ubiquitin conjugating enzyme E2 D2 [Source:HGNC Symbol;Acc:HGNC:12475] | 442 | 0.686 | 0.0824 | Yes |
| 6 | ERCC8 | "ERCC excision repair 8, CSA ubiquitin ligase complex subunit [Source:HGNC Symbol;Acc:HGNC:3439]" | 506 | 0.656 | 0.0967 | Yes |
| 7 | CDC20 | cell division cycle 20 [Source:HGNC Symbol;Acc:HGNC:1723] | 552 | 0.641 | 0.1120 | Yes |
| 8 | UBE2L6 | ubiquitin conjugating enzyme E2 L6 [Source:HGNC Symbol;Acc:HGNC:12490] | 873 | 0.550 | 0.1031 | Yes |
| 9 | HERC4 | HECT and RLD domain containing E3 ubiquitin protein ligase 4 [Source:HGNC Symbol;Acc:HGNC:24521] | 896 | 0.544 | 0.1173 | Yes |
| 10 | ELOB | elongin B [Source:HGNC Symbol;Acc:HGNC:11619] | 933 | 0.531 | 0.1300 | Yes |
| 11 | TRIM32 | tripartite motif containing 32 [Source:HGNC Symbol;Acc:HGNC:16380] | 993 | 0.519 | 0.1406 | Yes |
| 12 | KLHL9 | kelch like family member 9 [Source:HGNC Symbol;Acc:HGNC:18732] | 1006 | 0.516 | 0.1548 | Yes |
| 13 | ANAPC13 | anaphase promoting complex subunit 13 [Source:HGNC Symbol;Acc:HGNC:24540] | 1116 | 0.493 | 0.1607 | Yes |
| 14 | HERC3 | HECT and RLD domain containing E3 ubiquitin protein ligase 3 [Source:HGNC Symbol;Acc:HGNC:4876] | 1238 | 0.469 | 0.1649 | Yes |
| 15 | UBE2J1 | ubiquitin conjugating enzyme E2 J1 [Source:HGNC Symbol;Acc:HGNC:17598] | 1433 | 0.438 | 0.1626 | No |
| 16 | UBE2Q1 | ubiquitin conjugating enzyme E2 Q1 [Source:HGNC Symbol;Acc:HGNC:15698] | 1651 | 0.406 | 0.1575 | No |
| 17 | PRKN | parkin RBR E3 ubiquitin protein ligase [Source:HGNC Symbol;Acc:HGNC:8607] | 1972 | 0.364 | 0.1431 | No |
| 18 | RCHY1 | ring finger and CHY zinc finger domain containing 1 [Source:HGNC Symbol;Acc:HGNC:17479] | 2234 | 0.335 | 0.1325 | No |
| 19 | DDB1 | damage specific DNA binding protein 1 [Source:HGNC Symbol;Acc:HGNC:2717] | 2302 | 0.326 | 0.1369 | No |
| 20 | UBE4B | ubiquitination factor E4B [Source:HGNC Symbol;Acc:HGNC:12500] | 2367 | 0.318 | 0.1412 | No |
| 21 | SIAH1 | siah E3 ubiquitin protein ligase 1 [Source:HGNC Symbol;Acc:HGNC:10857] | 2389 | 0.315 | 0.1488 | No |
| 22 | UBE2D4 | ubiquitin conjugating enzyme E2 D4 (putative) [Source:HGNC Symbol;Acc:HGNC:21647] | 2610 | 0.290 | 0.1400 | No |
| 23 | HERC1 | HECT and RLD domain containing E3 ubiquitin protein ligase family member 1 [Source:HGNC Symbol;Acc:HGNC:4867] | 2684 | 0.281 | 0.1426 | No |
| 24 | UBE2B | ubiquitin conjugating enzyme E2 B [Source:HGNC Symbol;Acc:HGNC:12473] | 3028 | 0.247 | 0.1230 | No |
| 25 | WWP1 | WW domain containing E3 ubiquitin protein ligase 1 [Source:HGNC Symbol;Acc:HGNC:17004] | 3230 | 0.227 | 0.1139 | No |
| 26 | DDB2 | damage specific DNA binding protein 2 [Source:HGNC Symbol;Acc:HGNC:2718] | 3257 | 0.225 | 0.1185 | No |
| 27 | UBE2E1 | ubiquitin conjugating enzyme E2 E1 [Source:HGNC Symbol;Acc:HGNC:12477] | 3262 | 0.225 | 0.1247 | No |
| 28 | RNF7 | ring finger protein 7 [Source:HGNC Symbol;Acc:HGNC:10070] | 3264 | 0.225 | 0.1313 | No |
| 29 | SAE1 | SUMO1 activating enzyme subunit 1 [Source:HGNC Symbol;Acc:HGNC:30660] | 3423 | 0.209 | 0.1250 | No |
| 30 | UBE2I | ubiquitin conjugating enzyme E2 I [Source:HGNC Symbol;Acc:HGNC:12485] | 3424 | 0.209 | 0.1311 | No |
| 31 | UBA6 | ubiquitin like modifier activating enzyme 6 [Source:HGNC Symbol;Acc:HGNC:25581] | 3454 | 0.206 | 0.1349 | No |
| 32 | CUL3 | cullin 3 [Source:HGNC Symbol;Acc:HGNC:2553] | 3480 | 0.204 | 0.1389 | No |
| 33 | CUL2 | cullin 2 [Source:HGNC Symbol;Acc:HGNC:2552] | 3660 | 0.189 | 0.1305 | No |
| 34 | CDC23 | cell division cycle 23 [Source:HGNC Symbol;Acc:HGNC:1724] | 3729 | 0.183 | 0.1305 | No |
| 35 | NEDD4L | NEDD4 like E3 ubiquitin protein ligase [Source:HGNC Symbol;Acc:HGNC:7728] | 3733 | 0.183 | 0.1356 | No |
| 36 | CUL4B | cullin 4B [Source:HGNC Symbol;Acc:HGNC:2555] | 3783 | 0.179 | 0.1370 | No |
| 37 | UBE2NL | ubiquitin conjugating enzyme E2 N like (gene/pseudogene) [Source:HGNC Symbol;Acc:HGNC:31710] | 3917 | 0.163 | 0.1314 | No |
| 38 | KEAP1 | kelch like ECH associated protein 1 [Source:HGNC Symbol;Acc:HGNC:23177] | 4095 | 0.146 | 0.1218 | No |
| 39 | VHL | von Hippel-Lindau tumor suppressor [Source:HGNC Symbol;Acc:HGNC:12687] | 4142 | 0.143 | 0.1224 | No |
| 40 | UBE2G1 | ubiquitin conjugating enzyme E2 G1 [Source:HGNC Symbol;Acc:HGNC:12482] | 4294 | 0.129 | 0.1144 | No |
| 41 | UBE2Z | ubiquitin conjugating enzyme E2 Z [Source:HGNC Symbol;Acc:HGNC:25847] | 4375 | 0.123 | 0.1117 | No |
| 42 | MGRN1 | mahogunin ring finger 1 [Source:HGNC Symbol;Acc:HGNC:20254] | 4474 | 0.115 | 0.1074 | No |
| 43 | UBE2M | ubiquitin conjugating enzyme E2 M [Source:HGNC Symbol;Acc:HGNC:12491] | 4535 | 0.110 | 0.1060 | No |
| 44 | CDC16 | cell division cycle 16 [Source:HGNC Symbol;Acc:HGNC:1720] | 4566 | 0.108 | 0.1068 | No |
| 45 | WWP2 | WW domain containing E3 ubiquitin protein ligase 2 [Source:HGNC Symbol;Acc:HGNC:16804] | 4818 | 0.086 | 0.0897 | No |
| 46 | UBOX5 | U-box domain containing 5 [Source:HGNC Symbol;Acc:HGNC:17777] | 4882 | 0.081 | 0.0871 | No |
| 47 | UBE2A | ubiquitin conjugating enzyme E2 A [Source:HGNC Symbol;Acc:HGNC:12472] | 4973 | 0.075 | 0.0823 | No |
| 48 | BIRC2 | baculoviral IAP repeat containing 2 [Source:HGNC Symbol;Acc:HGNC:590] | 5054 | 0.069 | 0.0780 | No |
| 49 | PIAS3 | protein inhibitor of activated STAT 3 [Source:HGNC Symbol;Acc:HGNC:16861] | 5195 | 0.057 | 0.0687 | No |
| 50 | CDC34 | "cell division cycle 34, ubiqiutin conjugating enzyme [Source:HGNC Symbol;Acc:HGNC:1734]" | 5361 | 0.043 | 0.0571 | No |
| 51 | ANAPC10 | anaphase promoting complex subunit 10 [Source:HGNC Symbol;Acc:HGNC:24077] | 5424 | 0.039 | 0.0534 | No |
| 52 | UBE2L3 | ubiquitin conjugating enzyme E2 L3 [Source:HGNC Symbol;Acc:HGNC:12488] | 5428 | 0.038 | 0.0542 | No |
| 53 | UBA7 | ubiquitin like modifier activating enzyme 7 [Source:HGNC Symbol;Acc:HGNC:12471] | 5651 | 0.019 | 0.0374 | No |
| 54 | TRAF6 | TNF receptor associated factor 6 [Source:HGNC Symbol;Acc:HGNC:12036] | 5761 | 0.011 | 0.0292 | No |
| 55 | FANCL | FA complementation group L [Source:HGNC Symbol;Acc:HGNC:20748] | 5765 | 0.011 | 0.0293 | No |
| 56 | BIRC3 | baculoviral IAP repeat containing 3 [Source:HGNC Symbol;Acc:HGNC:591] | 5942 | -0.001 | 0.0156 | No |
| 57 | TRIM37 | tripartite motif containing 37 [Source:HGNC Symbol;Acc:HGNC:7523] | 6176 | -0.019 | -0.0021 | No |
| 58 | PML | PML nuclear body scaffold [Source:HGNC Symbol;Acc:HGNC:9113] | 6421 | -0.037 | -0.0201 | No |
| 59 | UBE3B | ubiquitin protein ligase E3B [Source:HGNC Symbol;Acc:HGNC:13478] | 6476 | -0.040 | -0.0231 | No |
| 60 | ELOC | elongin C [Source:HGNC Symbol;Acc:HGNC:11617] | 6556 | -0.046 | -0.0280 | No |
| 61 | HUWE1 | "HECT, UBA and WWE domain containing E3 ubiquitin protein ligase 1 [Source:HGNC Symbol;Acc:HGNC:30892]" | 6558 | -0.046 | -0.0267 | No |
| 62 | CUL4A | cullin 4A [Source:HGNC Symbol;Acc:HGNC:2554] | 6701 | -0.055 | -0.0362 | No |
| 63 | HERC2 | HECT and RLD domain containing E3 ubiquitin protein ligase 2 [Source:HGNC Symbol;Acc:HGNC:4868] | 6746 | -0.059 | -0.0379 | No |
| 64 | ANAPC5 | anaphase promoting complex subunit 5 [Source:HGNC Symbol;Acc:HGNC:15713] | 6751 | -0.059 | -0.0365 | No |
| 65 | ANAPC2 | anaphase promoting complex subunit 2 [Source:HGNC Symbol;Acc:HGNC:19989] | 6787 | -0.062 | -0.0374 | No |
| 66 | UBA3 | ubiquitin like modifier activating enzyme 3 [Source:HGNC Symbol;Acc:HGNC:12470] | 6936 | -0.075 | -0.0468 | No |
| 67 | FBXO2 | F-box protein 2 [Source:HGNC Symbol;Acc:HGNC:13581] | 6959 | -0.077 | -0.0463 | No |
| 68 | MAP3K1 | mitogen-activated protein kinase kinase kinase 1 [Source:HGNC Symbol;Acc:HGNC:6848] | 7066 | -0.085 | -0.0520 | No |
| 69 | UBE2N | ubiquitin conjugating enzyme E2 N [Source:HGNC Symbol;Acc:HGNC:12492] | 7099 | -0.087 | -0.0520 | No |
| 70 | CUL7 | cullin 7 [Source:HGNC Symbol;Acc:HGNC:21024] | 7105 | -0.088 | -0.0498 | No |
| 71 | UBA1 | ubiquitin like modifier activating enzyme 1 [Source:HGNC Symbol;Acc:HGNC:12469] | 7266 | -0.100 | -0.0594 | No |
| 72 | MDM2 | MDM2 proto-oncogene [Source:HGNC Symbol;Acc:HGNC:6973] | 7273 | -0.101 | -0.0569 | No |
| 73 | UBR5 | ubiquitin protein ligase E3 component n-recognin 5 [Source:HGNC Symbol;Acc:HGNC:16806] | 7285 | -0.101 | -0.0548 | No |
| 74 | CUL5 | cullin 5 [Source:HGNC Symbol;Acc:HGNC:2556] | 7314 | -0.103 | -0.0540 | No |
| 75 | NEDD4 | NEDD4 E3 ubiquitin protein ligase [Source:HGNC Symbol;Acc:HGNC:7727] | 7407 | -0.111 | -0.0579 | No |
| 76 | MID1 | midline 1 [Source:HGNC Symbol;Acc:HGNC:7095] | 7698 | -0.134 | -0.0767 | No |
| 77 | TRIP12 | thyroid hormone receptor interactor 12 [Source:HGNC Symbol;Acc:HGNC:12306] | 8290 | -0.178 | -0.1177 | No |
| 78 | UBE2K | ubiquitin conjugating enzyme E2 K [Source:HGNC Symbol;Acc:HGNC:4914] | 8386 | -0.185 | -0.1197 | No |
| 79 | BRCA1 | BRCA1 DNA repair associated [Source:HGNC Symbol;Acc:HGNC:1100] | 8417 | -0.187 | -0.1166 | No |
| 80 | SKP1 | S-phase kinase associated protein 1 [Source:HGNC Symbol;Acc:HGNC:10899] | 8454 | -0.190 | -0.1138 | No |
| 81 | SKP2 | S-phase kinase associated protein 2 [Source:HGNC Symbol;Acc:HGNC:10901] | 8625 | -0.203 | -0.1212 | No |
| 82 | XIAP | X-linked inhibitor of apoptosis [Source:HGNC Symbol;Acc:HGNC:592] | 8750 | -0.212 | -0.1247 | No |
| 83 | UBE3A | ubiquitin protein ligase E3A [Source:HGNC Symbol;Acc:HGNC:12496] | 8757 | -0.213 | -0.1189 | No |
| 84 | UBE3C | ubiquitin protein ligase E3C [Source:HGNC Symbol;Acc:HGNC:16803] | 8861 | -0.222 | -0.1205 | No |
| 85 | UBE2D1 | ubiquitin conjugating enzyme E2 D1 [Source:HGNC Symbol;Acc:HGNC:12474] | 8863 | -0.222 | -0.1141 | No |
| 86 | UBE4A | ubiquitination factor E4A [Source:HGNC Symbol;Acc:HGNC:12499] | 8939 | -0.227 | -0.1133 | No |
| 87 | CDC27 | cell division cycle 27 [Source:HGNC Symbol;Acc:HGNC:1728] | 8944 | -0.227 | -0.1069 | No |
| 88 | UBE2C | ubiquitin conjugating enzyme E2 C [Source:HGNC Symbol;Acc:HGNC:15937] | 9106 | -0.240 | -0.1125 | No |
| 89 | UBE2S | ubiquitin conjugating enzyme E2 S [Source:HGNC Symbol;Acc:HGNC:17895] | 9118 | -0.241 | -0.1063 | No |
| 90 | PPIL2 | peptidylprolyl isomerase like 2 [Source:HGNC Symbol;Acc:HGNC:9261] | 9148 | -0.244 | -0.1014 | No |
| 91 | CBL | Cbl proto-oncogene [Source:HGNC Symbol;Acc:HGNC:1541] | 9479 | -0.272 | -0.1193 | No |
| 92 | CBLC | Cbl proto-oncogene C [Source:HGNC Symbol;Acc:HGNC:15961] | 9553 | -0.279 | -0.1168 | No |
| 93 | ANAPC1 | anaphase promoting complex subunit 1 [Source:HGNC Symbol;Acc:HGNC:19988] | 9630 | -0.286 | -0.1144 | No |
| 94 | CUL1 | cullin 1 [Source:HGNC Symbol;Acc:HGNC:2551] | 9762 | -0.297 | -0.1159 | No |
| 95 | FZR1 | fizzy and cell division cycle 20 related 1 [Source:HGNC Symbol;Acc:HGNC:24824] | 9996 | -0.321 | -0.1248 | No |
| 96 | UBE2O | ubiquitin conjugating enzyme E2 O [Source:HGNC Symbol;Acc:HGNC:29554] | 10305 | -0.351 | -0.1386 | No |
| 97 | UBA2 | ubiquitin like modifier activating enzyme 2 [Source:HGNC Symbol;Acc:HGNC:30661] | 10360 | -0.357 | -0.1324 | No |
| 98 | BTRC | beta-transducin repeat containing E3 ubiquitin protein ligase [Source:HGNC Symbol;Acc:HGNC:1144] | 10836 | -0.409 | -0.1575 | No |
| 99 | UBE2D3 | ubiquitin conjugating enzyme E2 D3 [Source:HGNC Symbol;Acc:HGNC:12476] | 10967 | -0.427 | -0.1552 | No |
| 100 | UBE2W | ubiquitin conjugating enzyme E2 W [Source:HGNC Symbol;Acc:HGNC:25616] | 11238 | -0.462 | -0.1628 | No |
| 101 | PRPF19 | pre-mRNA processing factor 19 [Source:HGNC Symbol;Acc:HGNC:17896] | 11269 | -0.467 | -0.1515 | No |
| 102 | PIAS2 | protein inhibitor of activated STAT 2 [Source:HGNC Symbol;Acc:HGNC:17311] | 11288 | -0.470 | -0.1391 | No |
| 103 | RHOBTB2 | Rho related BTB domain containing 2 [Source:HGNC Symbol;Acc:HGNC:18756] | 11303 | -0.471 | -0.1264 | No |
| 104 | PIAS1 | protein inhibitor of activated STAT 1 [Source:HGNC Symbol;Acc:HGNC:2752] | 11422 | -0.490 | -0.1213 | No |
| 105 | FBXW7 | F-box and WD repeat domain containing 7 [Source:HGNC Symbol;Acc:HGNC:16712] | 11524 | -0.505 | -0.1144 | No |
| 106 | UBE2G2 | ubiquitin conjugating enzyme E2 G2 [Source:HGNC Symbol;Acc:HGNC:12483] | 11687 | -0.530 | -0.1116 | No |
| 107 | ITCH | itchy E3 ubiquitin protein ligase [Source:HGNC Symbol;Acc:HGNC:13890] | 11800 | -0.549 | -0.1043 | No |
| 108 | SMURF2 | SMAD specific E3 ubiquitin protein ligase 2 [Source:HGNC Symbol;Acc:HGNC:16809] | 11802 | -0.550 | -0.0882 | No |
| 109 | FBXW11 | F-box and WD repeat domain containing 11 [Source:HGNC Symbol;Acc:HGNC:13607] | 11982 | -0.590 | -0.0850 | No |
| 110 | PIAS4 | protein inhibitor of activated STAT 4 [Source:HGNC Symbol;Acc:HGNC:17002] | 12060 | -0.610 | -0.0731 | No |
| 111 | SMURF1 | SMAD specific E3 ubiquitin protein ligase 1 [Source:HGNC Symbol;Acc:HGNC:16807] | 12344 | -0.688 | -0.0751 | No |
| 112 | SOCS3 | suppressor of cytokine signaling 3 [Source:HGNC Symbol;Acc:HGNC:19391] | 12453 | -0.725 | -0.0623 | No |
| 113 | UBE2H | ubiquitin conjugating enzyme E2 H [Source:HGNC Symbol;Acc:HGNC:12484] | 12578 | -0.784 | -0.0491 | No |
| 114 | AIRE | autoimmune regulator [Source:HGNC Symbol;Acc:HGNC:360] | 12610 | -0.804 | -0.0279 | No |
| 115 | SOCS1 | suppressor of cytokine signaling 1 [Source:HGNC Symbol;Acc:HGNC:19383] | 12664 | -0.836 | -0.0076 | No |
| 116 | CBLB | Cbl proto-oncogene B [Source:HGNC Symbol;Acc:HGNC:1542] | 12725 | -0.887 | 0.0137 | No |
Table: GSEA details [plain text format]

  

Fig 2: KEGG\_UBIQUITIN\_MEDIATED\_PROTEOLYSIS      
 Blue-Pink O' Gram in the Space of the Analyzed GeneSet

  

Fig 3: KEGG\_UBIQUITIN\_MEDIATED\_PROTEOLYSIS: Random ES distribution      
 Gene set null distribution of ES for **KEGG\_UBIQUITIN\_MEDIATED\_PROTEOLYSIS**
